# Dual Band Metamaterial Antenna For LTE/Bluetooth/WiMAX System

**DOI:** 10.1038/s41598-018-19705-3

**Published:** 2018-01-19

**Authors:** Md. Mehedi Hasan, Mohammad Rashed Iqbal Faruque, Mohammad Tariqul Islam

**Affiliations:** 1 Space Science Center (ANGKASA), Universiti Kebangsaan Malaysia, 43600 Bangi, Malaysia; 2 Department of Electrical, Electronic and Systems Engineering, Universiti Kebangsaan Malaysia, 43600 Bangi, Malaysia

## Abstract

A compact metamaterial inspired antenna operate at LTE, Bluetooth and WiMAX frequency band is introduced in this paper. For the lower band, the design utilizes an outer square metallic strip forcing the patch to radiate as an equivalent magnetic-current loop. For the upper band, another magnetic current loop is created by adding metamaterial structure near the feed line on the patch. The metamaterial inspired antenna dimension of 42 × 32 mm^2^ compatible to wireless devices. Finite integration technique based CST Microwave Studio simulator has been used to design and numerical investigation as well as lumped circuit model of the metamaterial antenna is explained with proper mathematical derivation. The achieved measured dual band operation of the conventional antenna are sequentially, 0.561~0.578 GHz, 2.346~2.906 GHz, and 2.91~3.49 GHz, whereas the metamaterial inspired antenna shows dual-band operation from 0.60~0.64 GHz, 2.67~3.40 GHz and 3.61~3.67 GHz, respectively. Therefore, the metamaterial antenna is applicable for LTE and WiMAX applications. Besides, the measured metamaterial antenna gains of 0.15~3.81 dBi and 3.47~3.75 dBi, respectively for the frequency band of 2.67~3.40 GHz and 3.61~3.67 GHz.

## Introduction

With the fast development of the wireless communication technologies, having lightweight, low profile, superior performance, multi-band, low frequency band operation antennas are in good need to satisfy the increasing number of service bands, especially the Global system for mobile communication (GSM-900, GSM-1800), Global positioning system (GPS), Wireless LAN (WLAN) at 2.4 GHz and 5 GHz, Long term evolution (LTE) cover three bands, where the lower band includes frequency range of (698–966 MHz), middle band in the range of (1.427–2.69 GHz) and higher band in the range of (3.4–3.8 GHz). The Unlicensed National Information Infrastructure (U-NII) band is used in IEEE 802.11a devices and by different Internet Service Providers (ISPs). U-NII low (5.15–5.35 GHz) for WLAN operation, U-NII mid (5.47–5.725 GHz) for WiFi operation and U-NII high (5.725–5.875 GHz) for WLAN operations. Bluetooth works at (2.4–2.85 GHz) frequency, which is in ISM (Industrial, Scientific and Medical) band, for WiMax 2.3 GHz, 2.5 GHz, 3.5 GHz is used by Internet Service Provider in the United State and other countries. So, for satisfy the demand short distance wireless communication systems uses different methods, such as use the thickness of substrate materials, patches with multiple layers, insertion of slots/slits, use of metamaterial and modification of radiator shape have been observed to increases the performance of the antennas. Compared with the conventional antenna, the metamaterial antenna can effectively decrease the number of the antenna elements and enhance the performance of the antenna. At present, using metamaterial to enhance the performances of antenna, like, bandwidth, gain and efficiency is a demand of advance communication systems to support more user having lightweight, superior performance, multi-band, high data rate, etc. Nowadays, in the wireless communication a revolutionary has been created with the help of several communication systems, such as, 4 G mobile communication, Bluetooth, WLAN, WiFi, GPS, WiMAX, etc. Today the electronics system design industry have become increasingly focused on realising smaller electronic devices while maintaining or increasing the performances. Due to the effective and optimal performances metamaterial antennas are appearing as potential structures for future wireless communication systems.

Metamaterials are artificial materials with more compact in size compare with conventional material structures and have some infrequent properties, like negative refractive index, negative permeability, double negative characteristic, etc., which are not exist in natural materials. Therefore, due to these unique properties and for enhancing the performance of the antennas nowadays metamaterial antennas have become a research hotspot. Recently, a lots of dual- and triple-bands metamaterial (MMs) structure^1,2^ with metamaterial antennas have discussed in research articles. Metamaterial antenna is a class for antenna inspired by metamaterial structure on radiating patch or ground plane for better performances. A dual-band monopole antenna loaded with open complementary split-ring resonators (OCSRRs) covered the Bluetooth band (2.29~2.52 GHz) and the size of antenna was 40 × 30 × 1.5 mm^3^ was presented by Martínez *et al*.^3^. The antenna was designed by a rectangular monopole fed through a coplanar waveguide transmission line and loaded with an OCSRR. Further, an additional OCSRR was added with the proposed design for WiMAX band (3.56~3.78 GHz) and the gain of 1.4 and 1.7 dB for the Bluetooth and WiMAX applications^3^. A circularly polarised square slot (CPSS) antenna array composed of 2 × 2 CPSS for operating over the frequency band from 1 to 4.34 GHz (L- and S-band)^4^. A reconfigurable antenna for Bluetooth, WiMAX, and WLAN operations was presented, where the operating bands (2.2 to 2.53, 2.97 to 3.71, or 4.51 to 6.0 GHz) could be tuned by embedding pin diode on the antenna arm^5^. A metamaterial loaded rectangle monopole antenna (size of 45 × 40 mm^2^) showed resonance approximately, at 5.20 GHz for WLAN application and when an inverted-L slot was etched in antenna then the antenna produced a second resonance at around 4.10 GHz for WiMAX applications^6^. In^7^ a V-shaped 27 × 24 mm^2^ multi-band antenna is presented to operates at Bluetooth (2.40 to 2.48 GHz), WiMAX (3.30 to 3.70 GHz) and WLAN (5.15 to 5.35 and 5.73 to 5.85 GHz) applications. Further, a dual band antenna for 2.4/5.2 GHz WLAN operations was proposed, where dual band operation was achieved by utilizing stacking technique on a dog bone shaped 40 × 38 mm^2^ dipole antenna^8^. In addition, a simple compact antenna designed for the LTE (698–960 MHz) and WWAN (1710–2690 MHz) bands applications in^9^. Moreover, an antenna designed by a rectangular slot, a T-shaped feed patch, an inverted T-shaped stub and two E-shaped stubs to cover the frequency bands from (1.575 to 1.665 GHz) for the GPS, (2.4 to 2.545 GHz) for 802.11b&g WLAN, (3.27 to 3.97 GHz) for the WiMAX and (5.17 to 5.93 GHz) for the IEEE 802.11a WLAN systems^10^. In^11^ a dipole antenna was proposed that achieved dual-band operation at (2.37 to 2.82 GHz) and (3.14 to 4.10 GHz) for WLAN and WiMAX applications. Afterwards, in^12^ a multiband antenna introduced for GSM (880 to 960 and 1710 to 1880/1850 to 1990 MHz) and LTE 2300 (2300 to 2400 MHz) as well as LTE 2500 (2500 to 2690 MHz) applications, which peak gains were respectively, 2.12 and 3.82 dBi for the GSM and LTE bands. A compact monopole antenna consisted of a quarter-wavelength monopole, a ground plane and an annular sleeve have bandwidth of 160 MHz (from 2.38 to 2.54 GHz) and gain of <3.6 dBi for WLAN band operations^13^. However, in^14,15^ microstrip patch antennas were discussed for the mobile devices, such as GSM850/GSM900/UMTS/LTE and WLAN/WiMAX handsets. A single-layer rectangular patch with asymmetrical elliptical slot antenna was designed on the FR-4 dielectric material with 4.3 relative permittivity and tangent loss of 0.0027 was demonstrated by Abed *et al*. in 2016. The total dimension of the antenna was 70 × 50 mm^2^ and the asymmetrical slot is etched in the ground plate taking the form of the character ‘Q’. The resonance frequency bands from 2.4~2.56 GHz and 3.43~3.78 GHz was utilised for WiFi and WiMAX applications^16^. A compact MIMO antenna (Nandi *et al*.^17^), having size of 45 × 25 mm^2^ was designed by loading a modified square loop antenna with CRLH unit cell. The CRLH unit cell, consists of inter digital capacitor and two meandered inductive stubs. The MIMO antenna exhibited the bandwidth of 270 MHz (2.37~2.64 GHz) and 190 MHz (3.39~3.58 GHz) for WLAN and WiMAX applications. The gain of the antenna was −2 dBi and 0.14 dBi respectively from the frequency bands of 2.37~2.64 GHz and 3.39~3.58 GHz^17^. A frequency reconfigurable patch antenna was designed for lower (L-band) and upper (S-band), where the antenna utilizes a centre-fed patch with shorting rods at its edges for L-band and another magnetic current loop was created by adding four symmetrical resonant slots on the patch for the S-band as well as antenna bandwidths from 880 to 920 MHz and 1700 to 1820 MHz^18^. A direct-fed slot antenna was discussed, which larger slot generated a 0.5-wavelength resonant mode in the 2.4 GHz band in^19^. Besides, in 2017 a single open-slot antenna for LTE/WWAN operation was proposed for smartphone applications. The antenna efficiencies of <40% were demonstrated across the LTE/WWAN operation in the (698 to 960 MHz) and (1710 to 2690 MHz) bands^20^.

**Figure 1:**
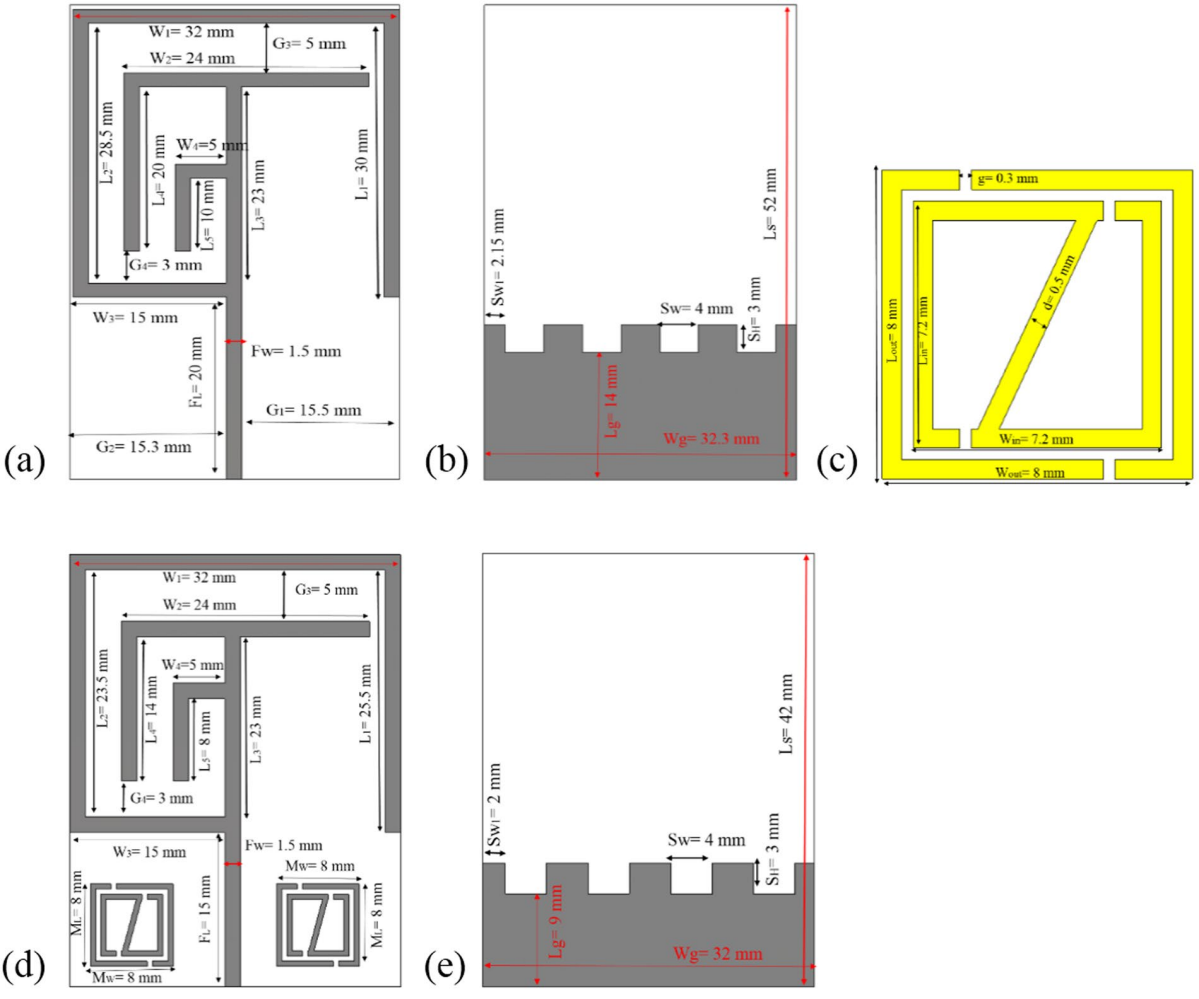
The geometry of the proposed: (**a**) Without MMs antenna front view, (**b**) Without MMs antenna back view, (**c**) Metamaterial (MMs) single unit cell, (**d**) With MMs antenna front view and (**e**) With MMs antenna back view.

The main concern of analysis of the above mentioned researches are to bring out and design a low profile, cost effective, lower frequency bands, better gain, stable radiation patterns and high efficiency metamaterial embedded antenna. Moreover, a dual-band metamaterial embedded antenna is proposed that is composed of a patch antenna with slotted ground plane. The integrated metamaterial structure is designed by combining the two ring resonator with an arrangement of splits and metal strips. A metal strips connected the upper and lower metal bar of the inner ring resonator and looks like a ‘Z-shaped’ structure. The proposed metamaterial antenna has been fabricated and measured. The measured results keep in good accordance with the simulated ones. Consequently, the simulated results of the proposed large size antenna without MMs in Fig. 1(a,b) achieve resonance peaks at 0.59, 2.55, and 3.17 GHz respectively that cover the bandwidth of 30, 590, 430 MHz as well as the measured −10 dB resonance peaks are respectively 0.56, 2.67, and 3.15 GHz, which cover the −10 dB bandwidth of 17 MHz (0.561~0.578 GHz), 560 MHz (2.346~2.906 GHz), and 580 MHz (2.91~3.49 GHz). In addition, after embedding the metamaterial structure in the antenna, antenna size becomes reduce displayed in Fig. 1(d,e) achieve a dual-band operation at 0.645~0.689 GHz (bandwidth of 44 MHz), 2.75~3.38 GHz (bandwidth of 630 MHz), and 3.45~3.56 GHz (bandwidth of 110 MHz), whereas the measured results resonance points are respectively, 0.63, 3.21, 3.63 GHz that covers the bandwidth of 40 MHz (0.60~0.64 GHz), 730 MHz (2.67~3.40 GHz), and 60 MHz (3.61~3.67 GHz) with the VSWR less than 2. As a result, without metamaterial embedded antenna applicable for LTE (0.561~0.578 GHz), Bluetooth (2.346~2.906 GHz), and WiMAX (2.91~3.49 GHz) applications as well as after embedding metamaterial the antenna size become compact and applicable for LTE (0.60~0.64 GHz), and WiMAX (2.67~3.40 GHz and 3.61~3.67 GHz) applications. Meanwhile, the stable gain, symmetrical and stable omnidirectional radiation patterns, low cross polarization and low back lobe are also obtained at the dual bands. In addition, the impacts on the performances of return loss (S_11_), cover bands, bandwidths, peak gains and total efficiencies by changing the antenna shapes, ground structures and the position of the embedded metamaterial are also analysed in detail.

The paper is structured as follows, Methods and measurement section starts with the step-by-step approach to design the metamaterial antenna, and adds symmetrical slots in ground plane to enhance the bandwidth. Later, this section discuss the simulation procedure with the proper mathematical explanation and extraction process of the effective permittivity, permeability, and refractive index of the embedded metamaterial structure. In addition, the lumped circuit model of the metamaterial and antenna structure as well as the measurement procedure of the proposed metamaterial antenna is explained elaborately. The results analysis is in results and discussion section. In this section, the performance of the different antenna structure without metamaterial, defective antenna ground plane are analyzed. Besides, the variation of the antenna performance is observed by embedding the metamaterial structure at different position of the antenna structure. However, then current distribution and the radiation pattern are also discussed in this section.

## Method and Measurement

Basis on the investigation on literature review, a low profile, multifunctional, small sized, low frequency bands without metamaterial antenna and with metamaterial antenna schematic geometries are shown in Fig. 1(a–e). The antennas have three layers; they are radiating patch, antenna substrate and ground plane as well as the design of antennas are done in millimeter (mm) scale. The presented 42 × 32 mm² metamaterial antenna is printed on a 1.60 mm thick (t) FR-4 material as substrate with relative permittivity 4.50 and loss tangent 0.02. On the back side, there is a ground plane (size of 12 × 32 mm²) including slots in the plane. Copper (conductivity of σ = 5 × 8 s/m and thickness of 0.035 mm) is used as the radiating patch and ground plane of the antennas. The feed line is 1.5 mm width and there is a little change of the width of the feed line makes a significant change in the resonances of the antennas. The feeding metal strip is connected to a 50Ω mini coaxial cable for testing the antenna. The integrated metamaterial structure is composed by combining the two ring resonator with an arrangement of splits and metal strips. A metal strips connected the upper and lower metal bar of the inner ring resonator and looks, like a ‘z-shape’ structure. While a single unit-cell dimension are set to 8.0 × 8.0 × 1.635 mm^3^ along the respective (*x*, *y*, *z*) axes. All the designs, simulations and investigation are done through the computer simulation technology (CST) Microwave Studio electromagnetic simulator tool. In addition, initially a patch is designed with a slotted ground for achieving low frequency resonance and then metamaterial are introduced to enhance the performances of the antenna with reduces of antenna size. However, the embedded metamaterial and slots in ground initial design equations are as follows,

1 $${M}_{W}\approx {M}_{L}\approx \frac{{L}_{S}}{5.25}\approx \frac{{W}_{S}}{4}$$

2 $${S}_{H}\approx \frac{{L}_{g}}{3}\approx \frac{{W}_{g}}{10.67}\approx \frac{{S}_{W}}{1.33}$$

where *M*_*W*_ and *M*_*L*_ are the length and width of the metamaterial unit cell in the patch, *S*_*H*_ is the length of slot, and *S*_*W*_ is the width of the slot in the ground plane.

**Figure 2:**
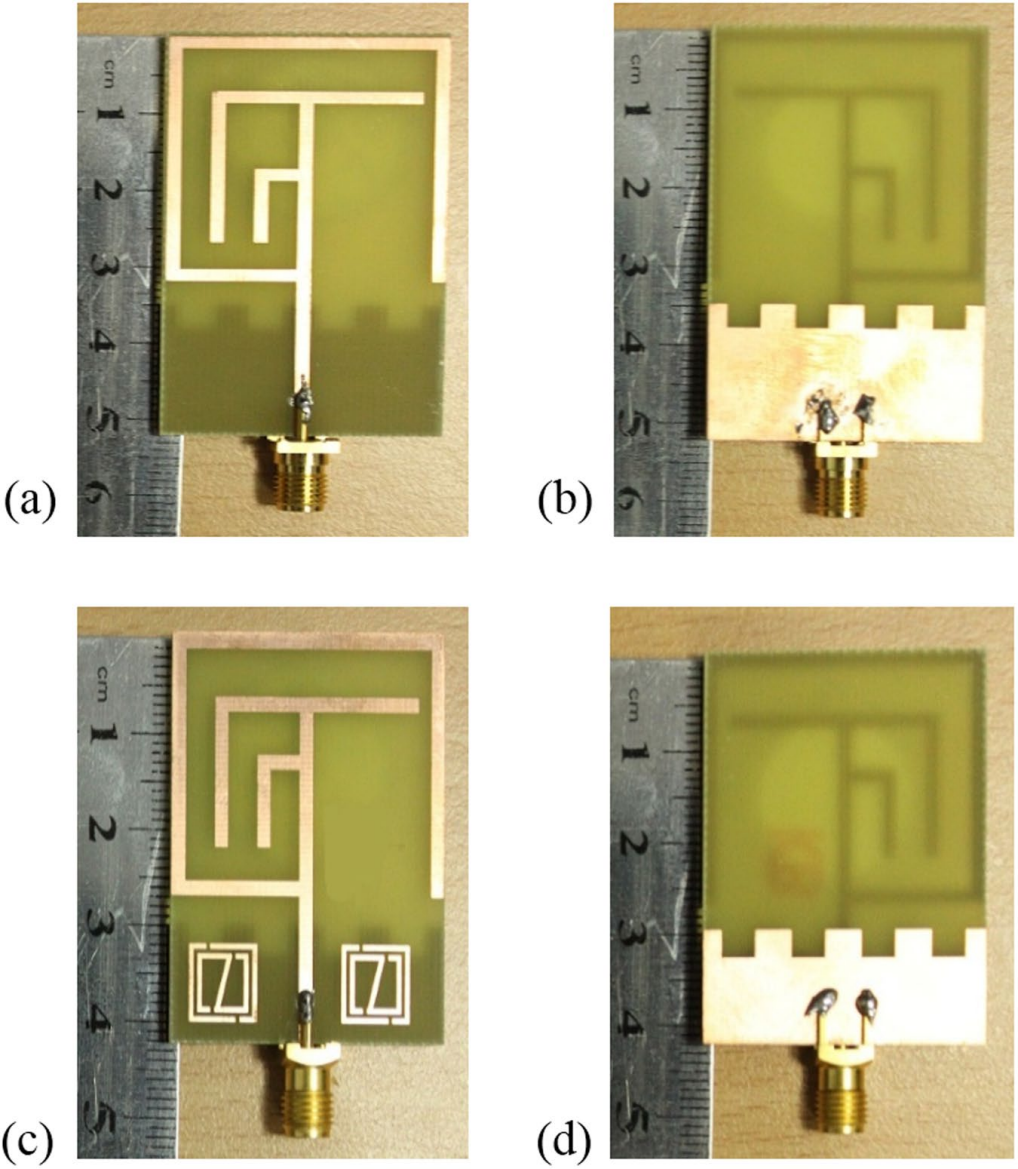
Fabricated geometry of the proposed: (**a**) Without MMs antenna front view, (**b**) Without MMs antenna back view, (**c**) With MMs antenna front view and (**d**) With MMs antenna back view.

The dimensions of the designed metamaterial (MMs) unit cell is, outer resonator length (L_out_) = 8.0 mm, outer resonator width (W_out_) = 8.0 mm, inner resonator length (L_IN_) = 7.20 mm, inner resonator width (W_IN_) = 7.20 mm, split gap (g) = 0.30 mm and resonator metal strips width (d) = 0.5 mm. Figure 2(a–d) illustrate the fabricated prototype of the proposed without metamaterial and with metamaterial integrated antennas. Moreover, Table 1 illustrates the design specification of the proposed MMs antenna.

**Table 1:** Design specification of the proposed metamaterial (MMs) antenna.

Parameters	L_1_	L_2_	L_3_	L_4_	L_5_	W_1_	W_2_	W_3_	W_4_
Size (mm)	25.5	23.5	23	14	8.0	32	24	15	5.0
Parameters	G_3_	G_4_	F_L_	F_W_	Lg	Wg	S_W_	S_H_	S_W1_
Size (mm)	5.0	3.0	15	1.5	9.0	32	4.0	3.0	2.0

On the basis of fundamental theoretical equations the mathematical formulae by which the design of proposed antenna was started can be derived as^21^,

3 $${\rm{The}}\,{\rm{effective}}\,{\rm{dielectric}}\,\mathrm{constant},\,{\in }_{{\rm{re}}}\approx \frac{{\in }_{{\rm{r}}}+1}{2}+\frac{{\in }_{{\rm{r}}}-1}{2}{(1+\frac{12{\rm{h}}}{{\rm{w}}})}^{-0.5}\,$$

4 $${\rm{Fringing}}\,{\rm{length}},\,{\rm{\Delta }}L\approx 0.412\{\frac{({\in }_{{\rm{re}}}+0.30)[\frac{w}{h}+0.26]}{({\in }_{{\rm{re}}}-0.258)[\frac{w}{h}+0.80]}\}h$$

5 $${\rm{Fundamental}}\,{\rm{frequency}},{f}_{1}\approx \frac{c}{2(L+2{\rm{\Delta }}L)\sqrt{{\in }_{{\rm{re}}}}}$$

Where ∈_*r*_ is the dielectric constant of FR-4 substrate, ${\in }_{r}$ = 4.40, ‘*w*’ is the width of the antenna element and ‘*h*’ is the height of the substrate material. The length and width of the antenna element is given by,

6 $${\rm{Patch}}\,{\rm{length}},\,L\approx \frac{{\lambda }_{0}}{2\sqrt{{\in }_{{\rm{r}}}}}-2{\rm{\Delta }}L\approx \frac{{c}_{0}}{2{f}_{0}\sqrt{{\in }_{{\rm{r}}}}}-2{\rm{\Delta }}L$$

7 $${\rm{Patch}}\,{\rm{width}},\,W\approx \frac{{\lambda }_{0}}{2}\sqrt{\frac{{\in }_{{\rm{r}}}+1}{2}}\approx \frac{{c}_{0}}{2{f}_{0}}\sqrt{\frac{{\in }_{{\rm{r}}}+1}{2}}$$

However, proper selection of feeding method, fine meshing of the structure, appropriate electromagnetic boundary conditions and more important is to right choice of driven solution are essentially required to obtain converged solution for the specified design. The expected performances in terms of return loss, gain, S-parameter, radiation patterns of the metamaterial embedded antenna have been investigated and numerically optimized with the help of electromagnetic simulation CST Microwave Studio, which generates the finite integration technique of Maxwell’s equations within boundary condition. To analyse the characteristics of the proposed metamaterial structure for embedded in the antenna structure reflection (*S*_*11*_) and transmission (*S*_*21*_) coefficients are extracted to calculate the effective permittivity (*ε*_*r*_), permeability (*μ*_*r*_), and refractive index (*n*_*r*_) are expressed as follows^22,23^,

8 $${{\rm{V}}}_{1}={{\rm{S}}}_{21}+{{\rm{S}}}_{11}$$

9 $${{\rm{V}}}_{2}={{\rm{S}}}_{21}-{{\rm{S}}}_{11}$$

10 $$\begin{array}{c}{\varepsilon }_{r}\approx \frac{2}{j{k}_{0}d}\times \frac{(1-{V}_{1})}{(1+{V}_{1})}\\ Effective\,Permittivity,{\varepsilon }_{r}\approx \frac{c}{j\pi fd}\times \{\frac{(1-{S}_{21}-{S}_{11})}{(1+{S}_{21}+{S}_{11})}\}\end{array}$$

11 $$\begin{array}{c}{\mu }_{r}\approx \frac{2}{j{k}_{0}d}\times \frac{(1-{V}_{2})}{(1+{V}_{2})}\\ Effective\,Permeability,{\mu }_{r}\approx \frac{c}{j\pi fd}\times \{\frac{(1-{S}_{21}+{S}_{11})}{(1+{S}_{21}-{S}_{11})}\}\end{array}$$

12 $$\begin{array}{c}{n}_{r}\approx \sqrt{{\varepsilon }_{r}{\mu }_{r}}\\ Refractive\,Index,{n}_{r}\approx \frac{c}{j\pi fd}\sqrt{\{\frac{{({S}_{21}-1)}^{2}-{S}_{11}^{2}}{{({S}_{21}+1)}^{2}-{S}_{11}^{2}}\}\,}\end{array}$$

**Figure 3:**
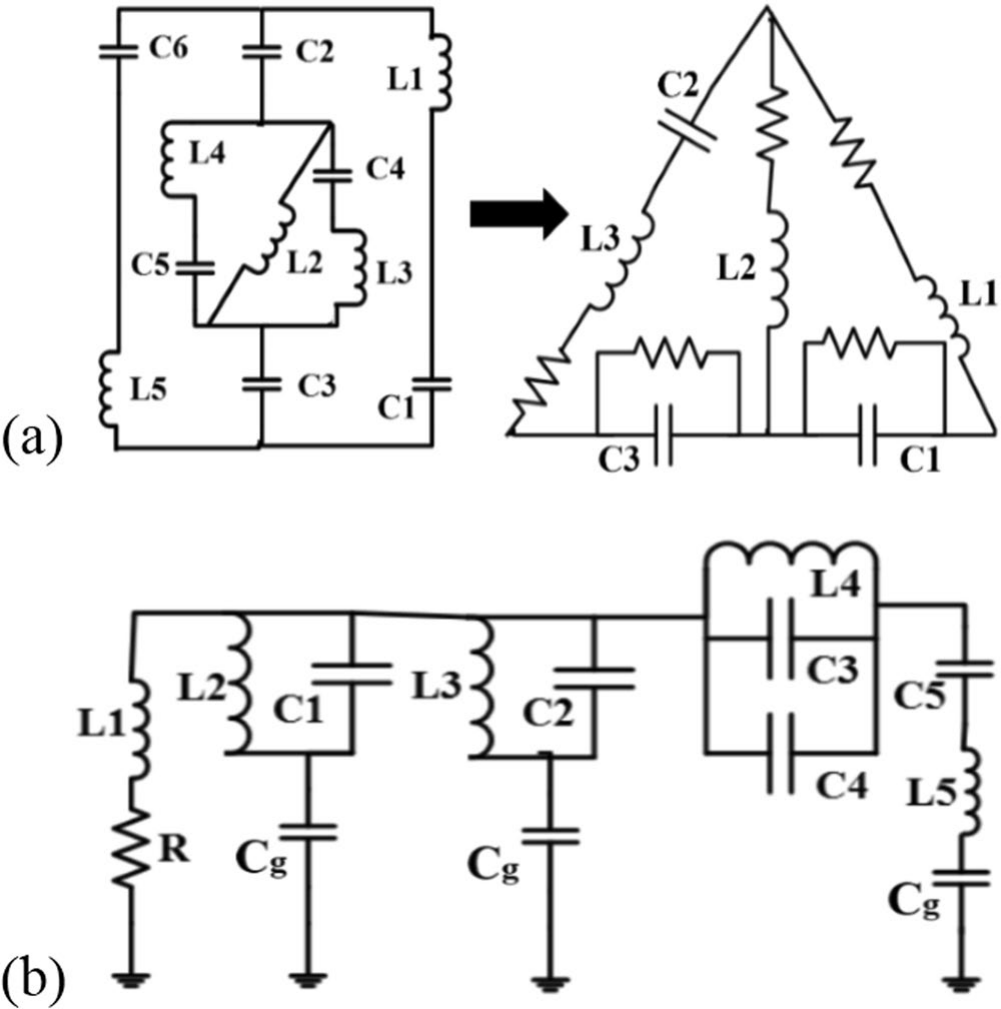
Lumped element equivalent circuit model of the designed: (**a**) Metamaterial unit cell structure (whereas the values of the inductor are respectively, L_1 _= 220 μH, L_2 _= 2.2 nH, L_3_ = 1.1 nH and the capacitance are sequentially, C_1_ = 5.6 pF, C_2_ = 1.5 pF, C_3_ = 0.51 pF) and (**b**) Proposed antenna structure (inductors are respectively, L_1 _= 6.2 nH, L_2 _= 4.3 nH, L_3 _= 3.3 nH, L_4 _= 4.7 nH, L_5 _= 5.6 nH and capacitors are sequentially, C_1_ = 0.1 pF, C_2_ = 0.16 pF, C_3 _= 0.15 pF, C_4_ = 0.20 pF, C_5_=4.7 pF).

The lumped equivalent circuit model^24,25^ of designed metamaterial unit cell and the metamaterial antenna are shown in Fig. 3(a,b), in the circuit model the combination of inductors and capacitors both in series and parallel. For equivalent circuit, we included an inductor for the metal strip and capacitors for the splits or gaps. The increase in the number of split rings will increase the number of split gaps and metallization on the substrate; thus, an increase in the surface electric field will be observed on the split gap areas and overall surface. The increasing values of overall capacitance, which includes gap and surface capacitance, will reduce the operating frequency as they are inversely proportional to each other. The strips form the magnetic inductance and can be considered as inductors. The capacitance is mainly formed in and around split gap areas. The split ring resonator exhibit electromagnetic resonance when the electric energy stored in capacitor; i.e., gap is in balance with the magnetic energy stored in the inductors, i.e., strips. The changes in capacitance ‘*C’* and inductance ‘*L’* due to dielectric loading leads to a considerable shift in the frequency of resonance.

**Figure 4:**
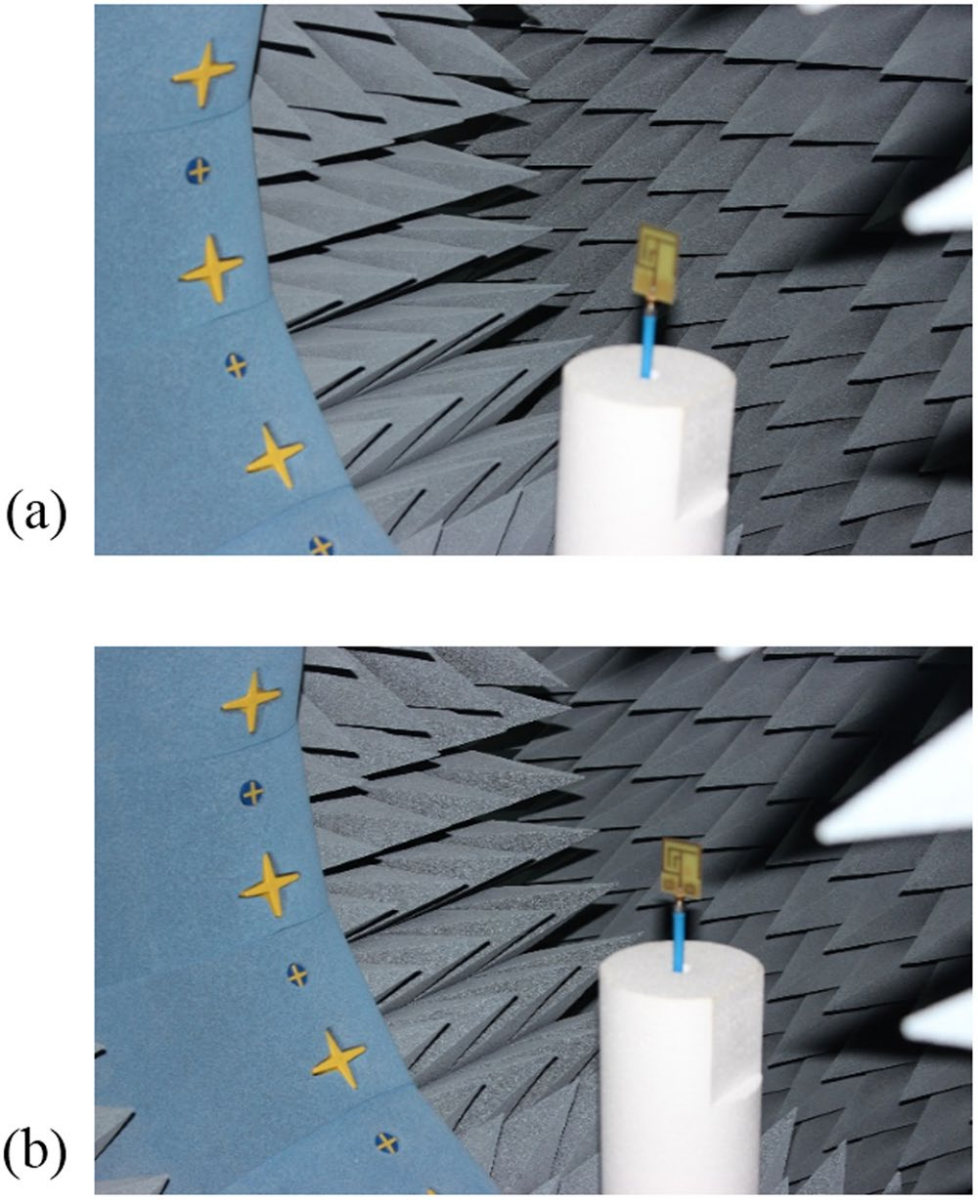
Measurement in the Satimo StarLab: (**a**) Without MMs antenna and (**b**) With MMs antenna.

To examine the actual performance and validate the simulation results of without MMs antenna and metamaterial antenna are fabricated on epoxy resin fibre substrate material. An Agilent N5227A vector network analyser (VNA) with a range of up to 20 GHz is used to scattering parameters, VSWR measurement. Figure 2(a–d) show the photograph of proposed antenna prototypes. In the simulated and measured results of the compact without MMs antenna and with MMs antennas a slight discrepancy occurred and that led to the differences between simulated and measured return loss of the proposed metamaterial antenna. Satimo StarLab near-field antenna measurement system with a range of 800 MHz to 18 GHz as shown in Fig. 4(a,b), which is used to measure the gain, efficiency and radiation pattern characteristics. This system allows measuring the antenna’s electric fields within the near-field region with an aim to compute the corresponding far-field values of the antenna under test (AUT). The AUT, placed on the test board, is positioned in the middle of a circular “arch” that contains 16 individual measuring probes. These probes are placed at equal distance surrounding the circular surface. The AUT is rotates horizontally in 360° angle, and this rotation and array of probes together do a full 3D scan of AUT and collecting data for 3D radiation patterns. The far-field data then is employed to compute the gain and efficiency of the AUT.

## Results and Discussions

**Figure 5:**
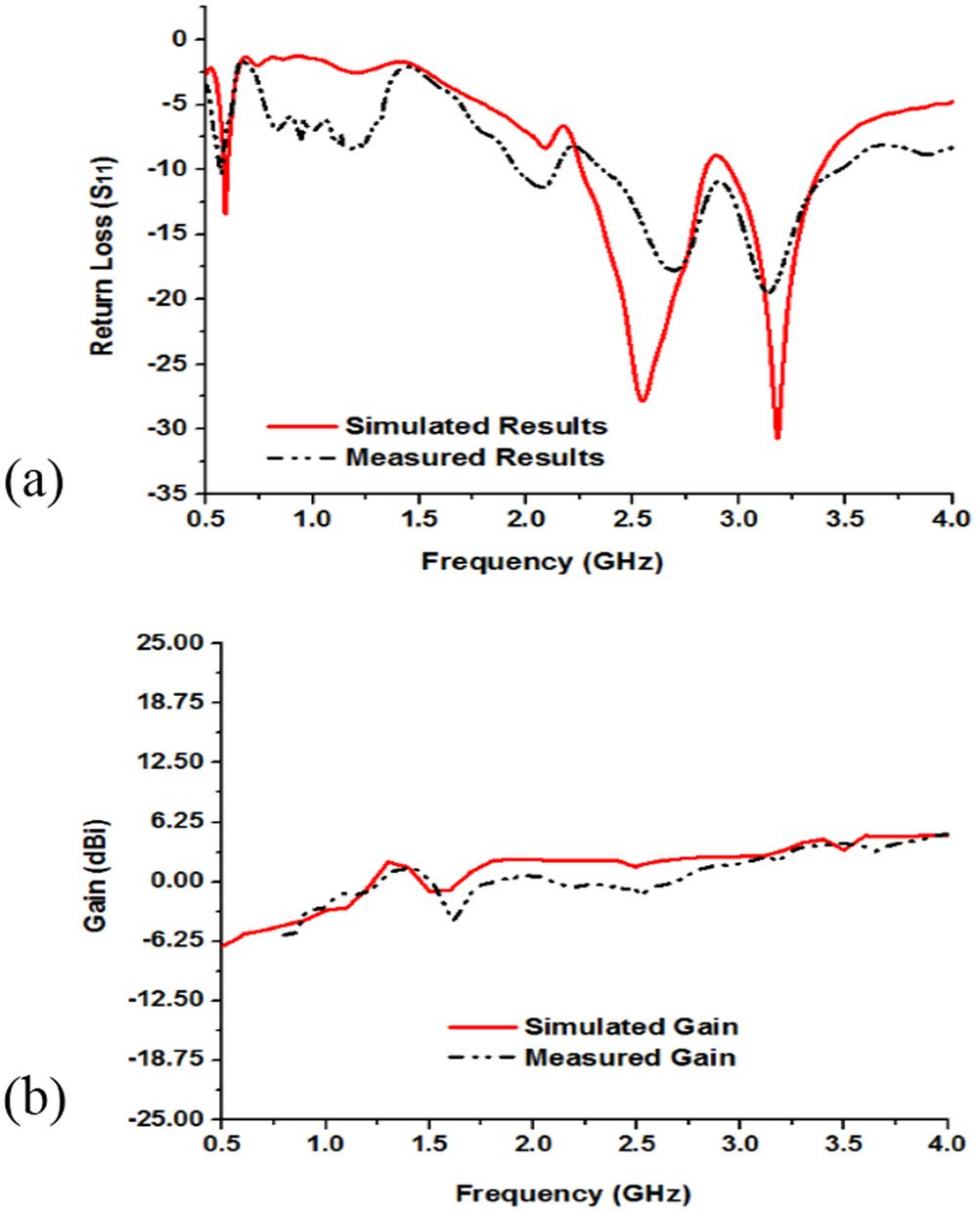
Measured and simulated: (**a**) Return loss (S_11_) and (**b**) Gain of the without MMs antenna shown in Fig. 2(a,b).

Figure 5(a,b) show the measured and simulated results of the fabricated antenna (in Fig. 2(a,b)). From Fig. 5(a) the simulated results of the antenna without metamaterial exhibit the resonance peaks at 0.59, 2.55, and 3.17 GHz that cover the bandwidth of 30, 590, 430 MHz respectively as well as the measured resonance peaks are respectively 0.56, 2.67, and 3.15 GHz, which cover the bandwidth of 20 MHz (from 0.56~0.58 GHz), 560 MHz (from 2.35~2.91 GHz), and 580 MHz (from 2.91~3.49 GHz). So, the antenna is applicable for LTE (0.561~0.578 GHz), Bluetooth (2.346~2.906 GHz), and WiMAX (2.91~3.49 GHz) applications. The measured and simulated gain of the antenna are shown in Fig. 5(b). It can be seen that the measured gain from 2.346 to 2.906 GHz and 2.91 to 3.49 GHz are respectively 0.05~2.70 dBi and 2.73~4.24 dBi, whereas the simulated gain from 2.26 to 2.84 GHz and 2.95 to 3.38 GHz are sequentially from 2.0~2.68 dBi and 2.89~4.54 dBi. However, in Fig. 5(b) the measured gain frequency range is from 0.8 GHz to 4.0 GHz, whereas the simulated frequency ranges from 0.5 GHz to 4.0 GHz because the measurement is done is Satimo StarLab and the Satimo StarLab can measured from 0.8 GHz to 18.0 GHz.

**Figure 6:**
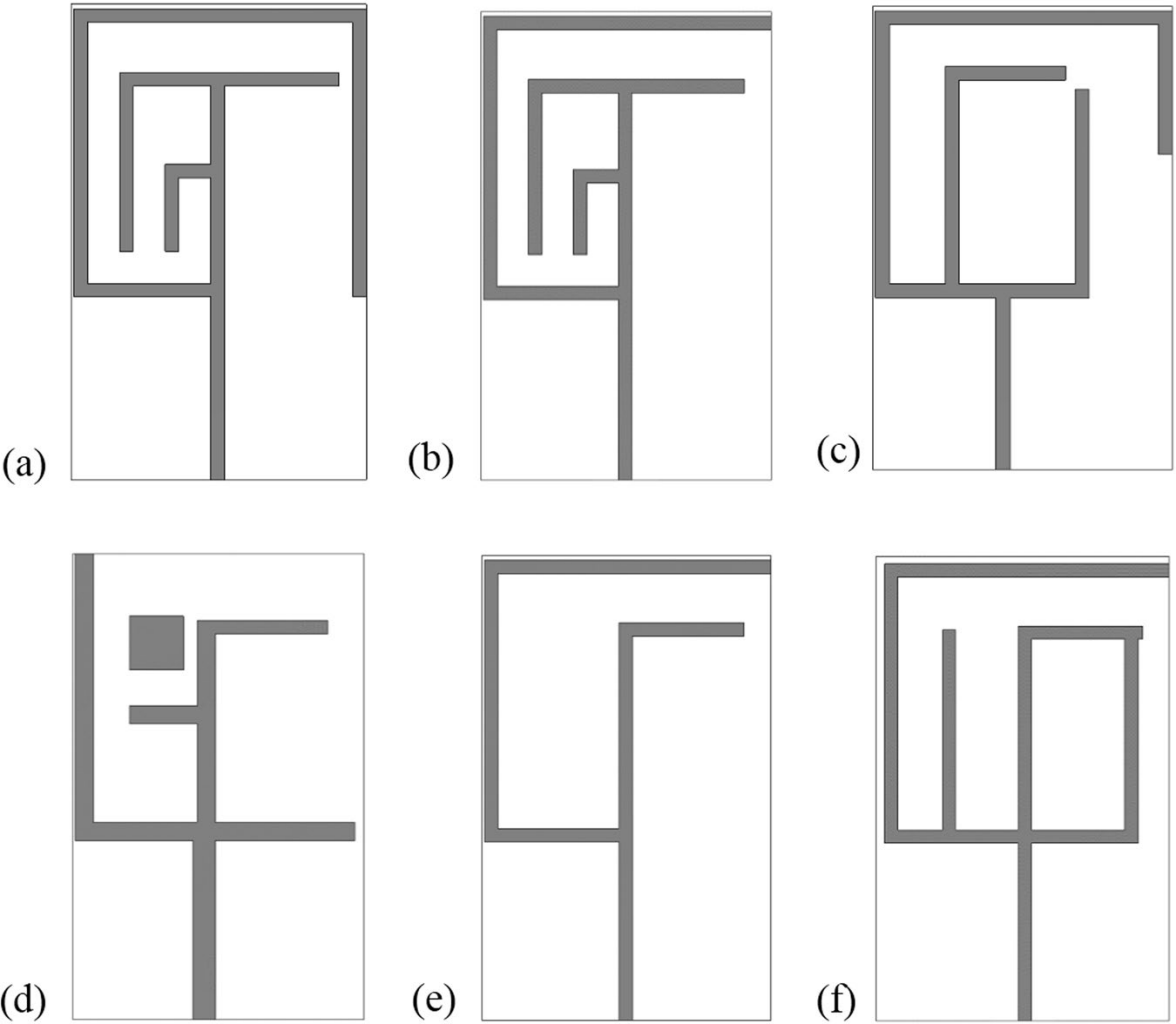
Parametric analysis of the designed different without MMs antenna geometry: (**a**) Antenna-1, (**b**) Antenna-2, (**c**) Antenna-3, (**d**) Antenna-4, (**e**) Antenna-5 and (**f**) Antenna-6.

In the antenna configuration the inductive effect is formed by the metallic part and the capacitive effect is induced by the slots, gaps, or splits. The raise of the inductive and capacitive effect minimizes together. Because, if the inductance raise resonance points shifts toward lower frequency and the resonance points shift to higher frequency for the increase of capacitive effect. However, the inductance and capacitance describes the resonances point, which depend on the design specifications of the antenna structure. The variation of any geometrical parameters corresponding to the change of the inductances and capacitances, causes the shift of the resonant frequencies. The numerical study on different shape antennas without embedded the metamaterial structure are seen in Fig. 6(a–f).

**Figure 7:**
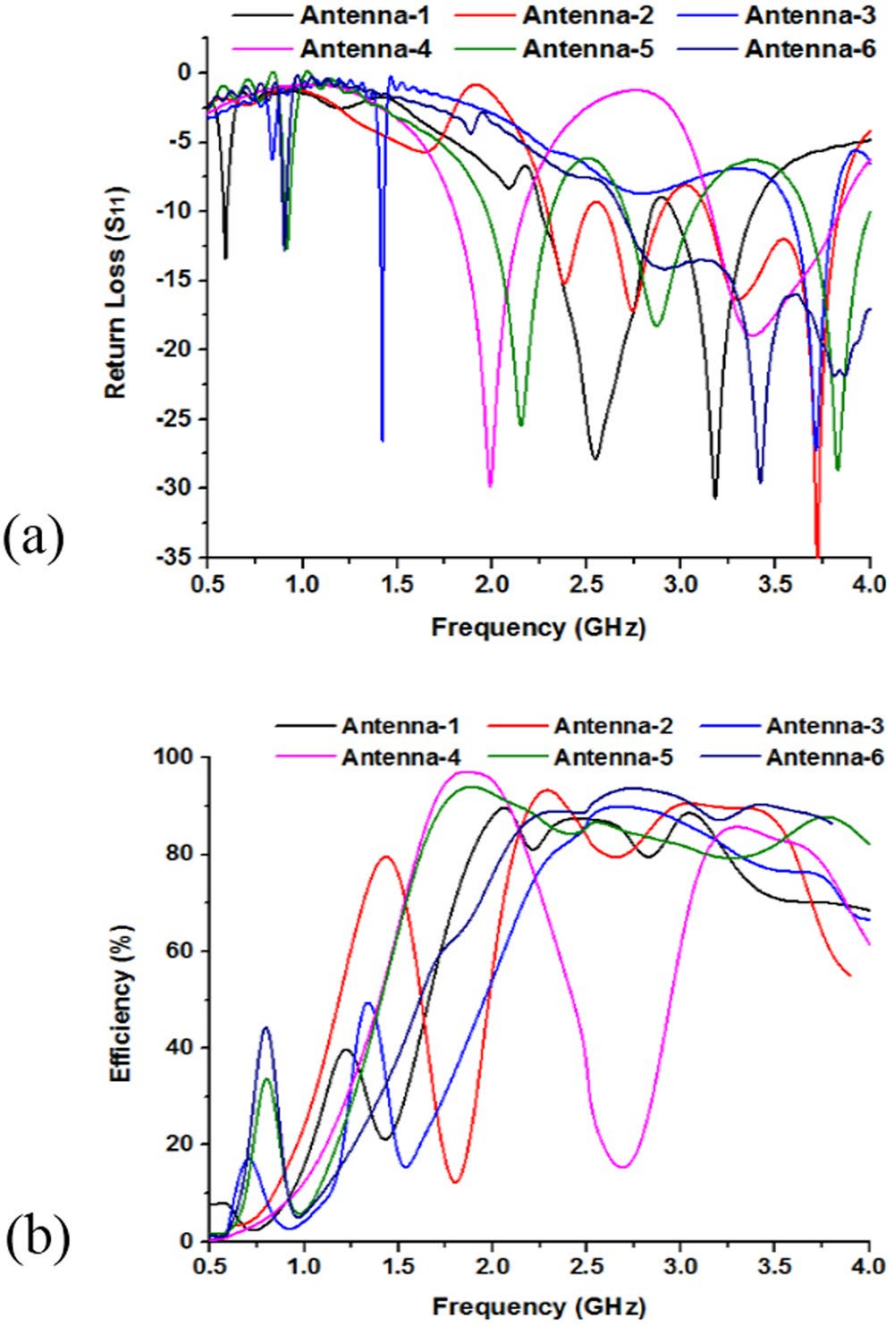
Performance analysis of designed different without MMs antenna geometry by: (**a**) Return loss (S_11_) and (**b**) Efficiency.

Figure 7(a,b) and Table 2 describes the variation of the return loss (*S*_*11*_), bandwidth and efficiency with the change of the antenna structures. It is seen that antenna-1 has three resonance points that cover the L- and S-bands, whereas the bandwidths of the resonance bands are respectively 30 MHz, 590 MHz, and 430 MHz. Similarly, antenna-5 has the similar three resonance peaks sequentially, 0.91 GHz, 2.15 GHz, 3.83 GHz but the covered bandwidth are smaller than the antenna-1 configuration. Moreover, reduction of the strips lines in the antenna-5 configuration reducing the current path and metallic portion of the antenna than antenna-1 structure, which make variation of these two antenna configurations. It is also observer that high frequency performance can be improved employing square metallic patch near the feed line in Fig. 6(d) and rearrange metal bar in antenna structure in Fig. 6(b,c).

**Table 2:** Performances analysis of the designed antenna configurations.

Antenna Configurations	Antenna-1	Antenna-2	Antenna-3	Antenna-4	Antenna-5	Antenna-6
Resonant Frequency (GHz)	0.59, 2.55, 3.17	2.38, 2.73, 3.72	1.42,3.72	1.98, 3.36	0.91, 2.15, 3.83	0.90, 3.41
Band covered	L-, S-Band	S-Band	S-Band	L-, S-Band	L-, S-Band	L-, S-Band
Bandwidth (MHz)	30, 590, 430	220, 270, 710	40, 230	380, 680	40, 340, 350	30, 1310
Efficiency	91%	94%	90%	97%	94%	93%

Usually, a large metal strips or slots in antenna structure is used to achieve a high level electromagnetic coupling to antenna structural arrangement. Therefore, variation of antenna structure change the coupling and control of impedance matching. It is found that by enhancing the coupling between the antenna radiators and feed line, good impedance matching can be obtained. If the coupling increases from optimum value impedance matching becomes poor. Figure 7(b) show the efficiency of the antenna configurations, where the highest efficiency is recorded as 97% with an average of 93%. It is quite visible from surface current that current are concentrated around the feeding point at the antenna and flow in the antenna patch. Moreover, the efficiency of the (antenna-1 to antenna-6) configurations are respectively, 91%, 94%, 90%, 97%, 94%, and 93%.

**Figure 8:**
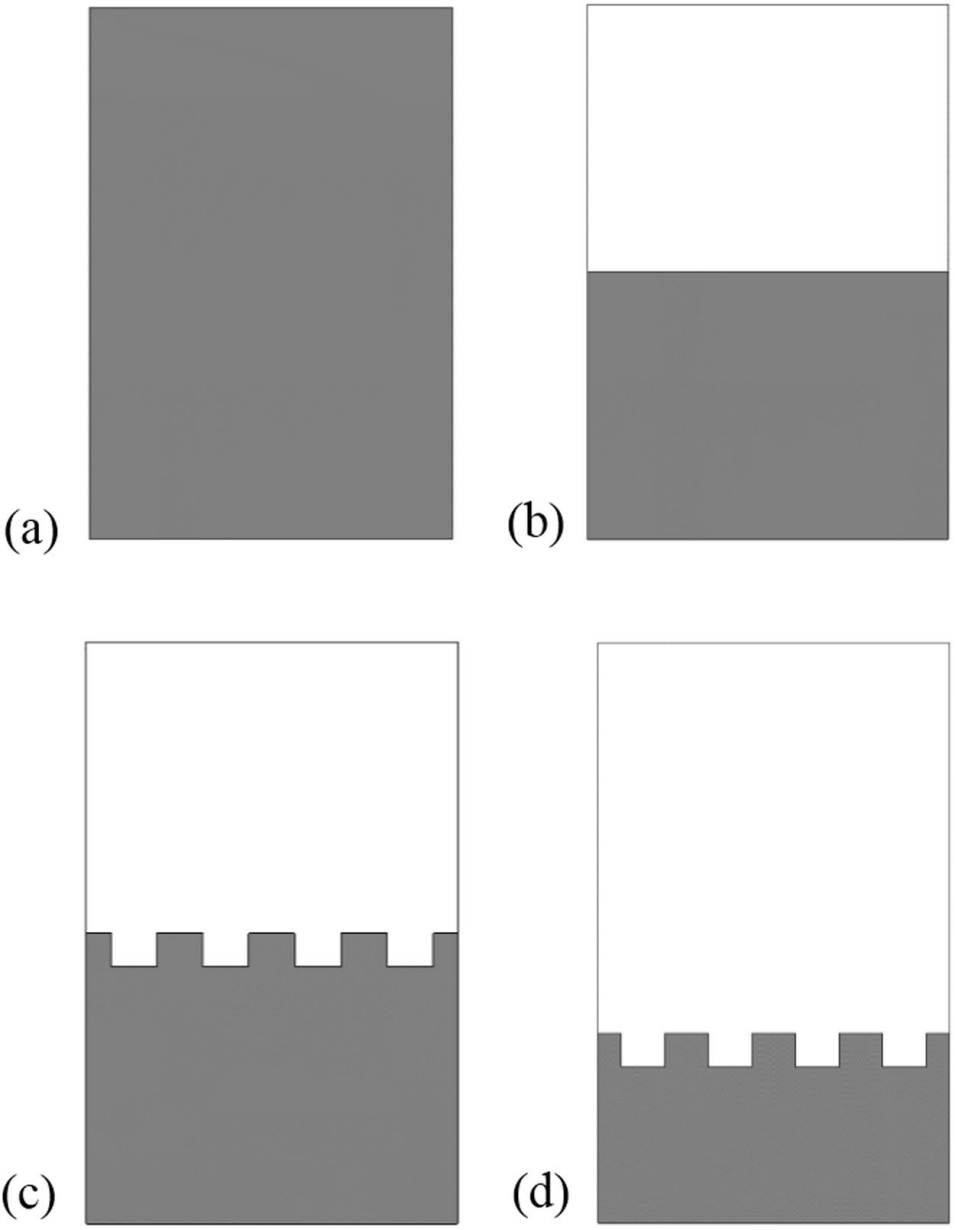
Parametric analysis of the without MMs antenna by: (**a**) Full ground plane, (**b**) Half ground plane, (**c**) Half slotted ground and (**d**) Slotted ground plane.

Figure 8(a–d) show the full-, half-, and slotted-ground plane structure of the proposed antenna. The ground plane has the effect on the antenna bandwidth. Bandwidth is changed basis on the ground plane length and width. At lower frequency less than the resonance frequency, the ground behaves like an inductor and at higher frequencies it behaves like capacitor. The slots on the ground is electrically coupled to the patch through the line capacitance, whereas the magnetic coupling can be modeled by the mutual inductance between the patch and inductor.

**Figure 9:**
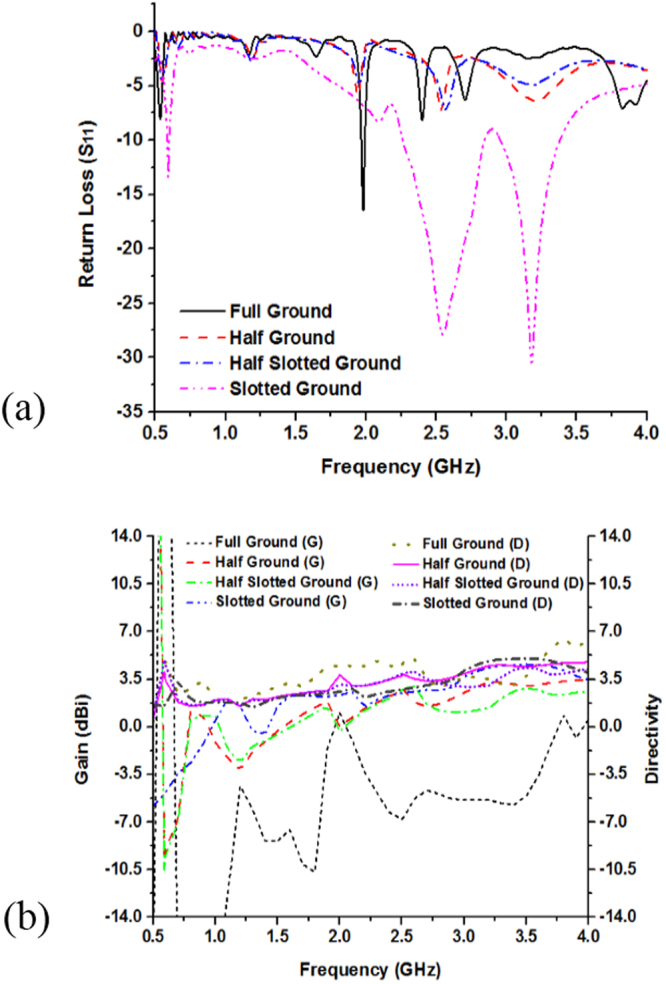
Performance analysis of the proposed without MMs antenna various ground plane structure by: (**a**) Return loss (S_11_) and (**b**) Gain & Directivity.

Figure 9(a) show the return loss and bandwidth as well as Fig. 9(b) shows gain and directivity effect of modifying the ground plane of the antenna. The results show that while increasing the length of the ground plane, the bandwidth will decreased because the capacitance is increased when the ground length increased. So, it is found that the bandwidths of the antenna are significantly affected by the ground plane length. Moreover, Fig. 9(b) show parametric analysis on gain and directivity of the full-length, half-length, half-slotted and slotted ground plane antennas. At 1.98 GHz the full-length ground plane antenna have gain 1.01 dBi. Further, resonance peaks at 2.53 GHz gain and directivity of the half-length ground antenna are respectively, 2.72 dBi and 3.78 dBi whereas at 2.58 GHz half-length slotted grounded antenna have the gain of 2.98 dBi and directivity of 4.05 dBi. Moreover, the slotted ground antenna (in Fig. 8(d)) has the larger gain and the directivity than the full-length, half-length plane and slotted ground plane antenna. At 3.17 GHz the slotted ground plane antenna (in Fig. 8(d)) have gain of 4.23 dBi and directivity 4.79 dBi in Fig. 9(b).

### Results analysis of metamaterial antenna

**Figure 10:**
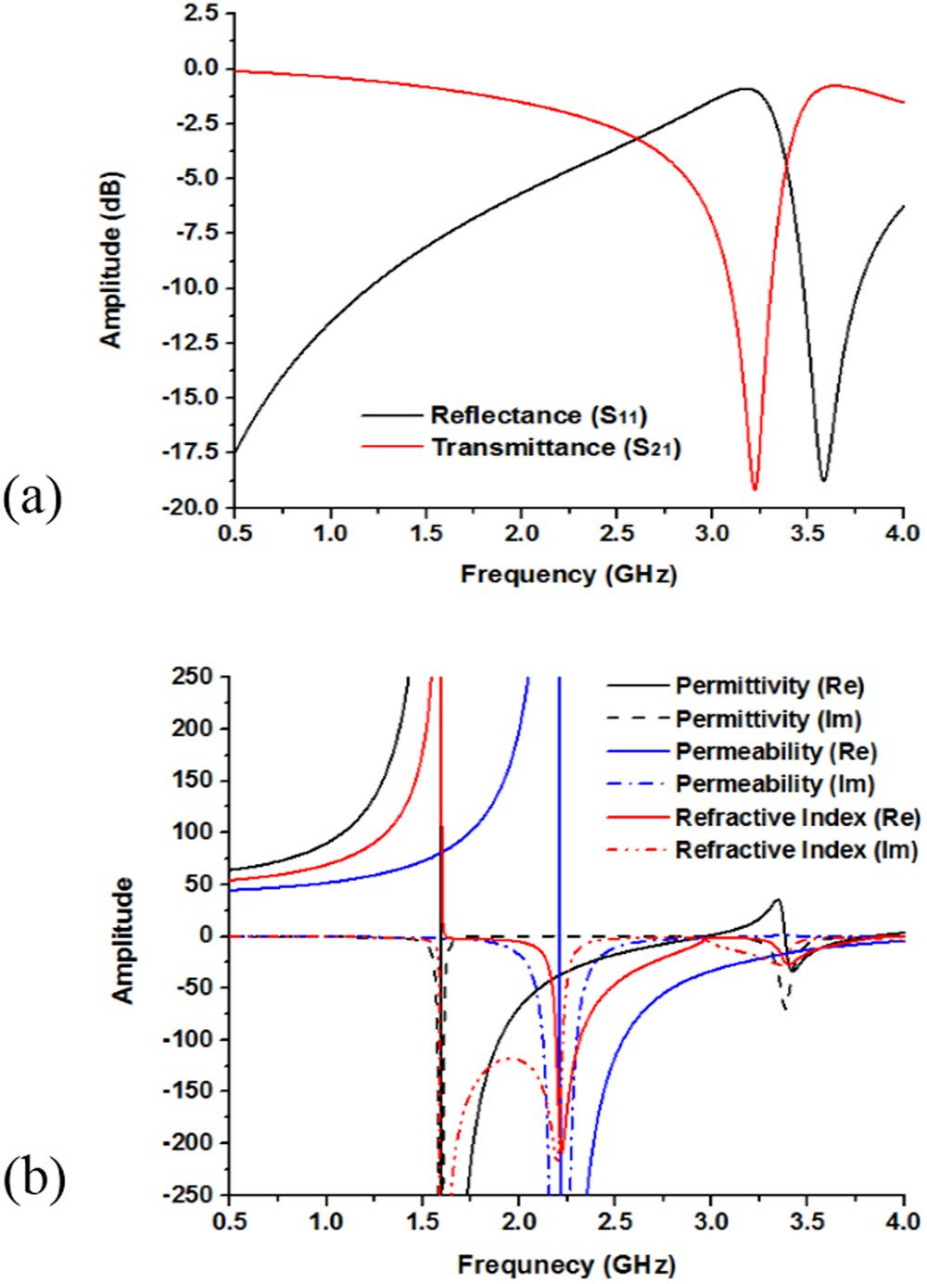
(**a**) Scattering parameters and (**b**) Permittivity, permeability and refractive index of the embedded left-handed metamaterial structure.

The electromagnetic characteristics of the metamaterial can be described by the reflection (S_11_) and transmission (S_21_) coefficients. From Fig. 10(a), the result of transmittance resonance at 3.18 GHz (amplitude of −19 dB) and the reflectance at 3.55 GHz (amplitude of −18.25 dB). The effective permittivity, permeability and refractive index of the embedded metamaterial structure are shown in Fig. 10(b), where the negative permeability is achieved from 2.21 to 4.0 GHz. In addition, the negative permittivity is achieved at two frequency ranges. The first one is approximately from 1.59 to 2.96 GHz, while the second one is from 3.38 to 3.83 GHz. There is variation between the results of permittivity and permeability for the polarization effects on the internal structure of the materials. Moreover, refractive index exhibits two major negative frequency range. The first one is around from 1.63 to 3.06 GHz and the last one is from 3.09 to 3.85 GHz, where the covers bandwidth for those frequency ranges are respectively, 1.43 GHz and 0.76 GHz. Moreover, according to the left handed characteristics, if the permittivity and permeability are simultaneously negative, then the refractive index will be negative. Therefore, from the frequency ranges 2.21 to 2.97 GHz and 3.38 to 3.84 GHz the effective permittivity, permeability and refractive index show the negative magnitude. As a result, the metamaterial structure is called as a left handed metamaterial for any frequency points in 2.21 to 2.97 GHz and 3.38 to 3.84 GHz frequency range. Moreover, at 3.51 GHz transmittance (S_21_) resonance point, designed metamaterial permittivity, permeability and refractive index are −17.24, −12.72 and −15.07 respectively shown in Table 3.

**Table 3:** Left-handed characteristics of the embedded metamaterial structure.

Transmittance Resonance	Permittivity	Permeability	Refractive Index
3.51 GHz	−17.24	−12.72	−15.07

**Figure 11:**
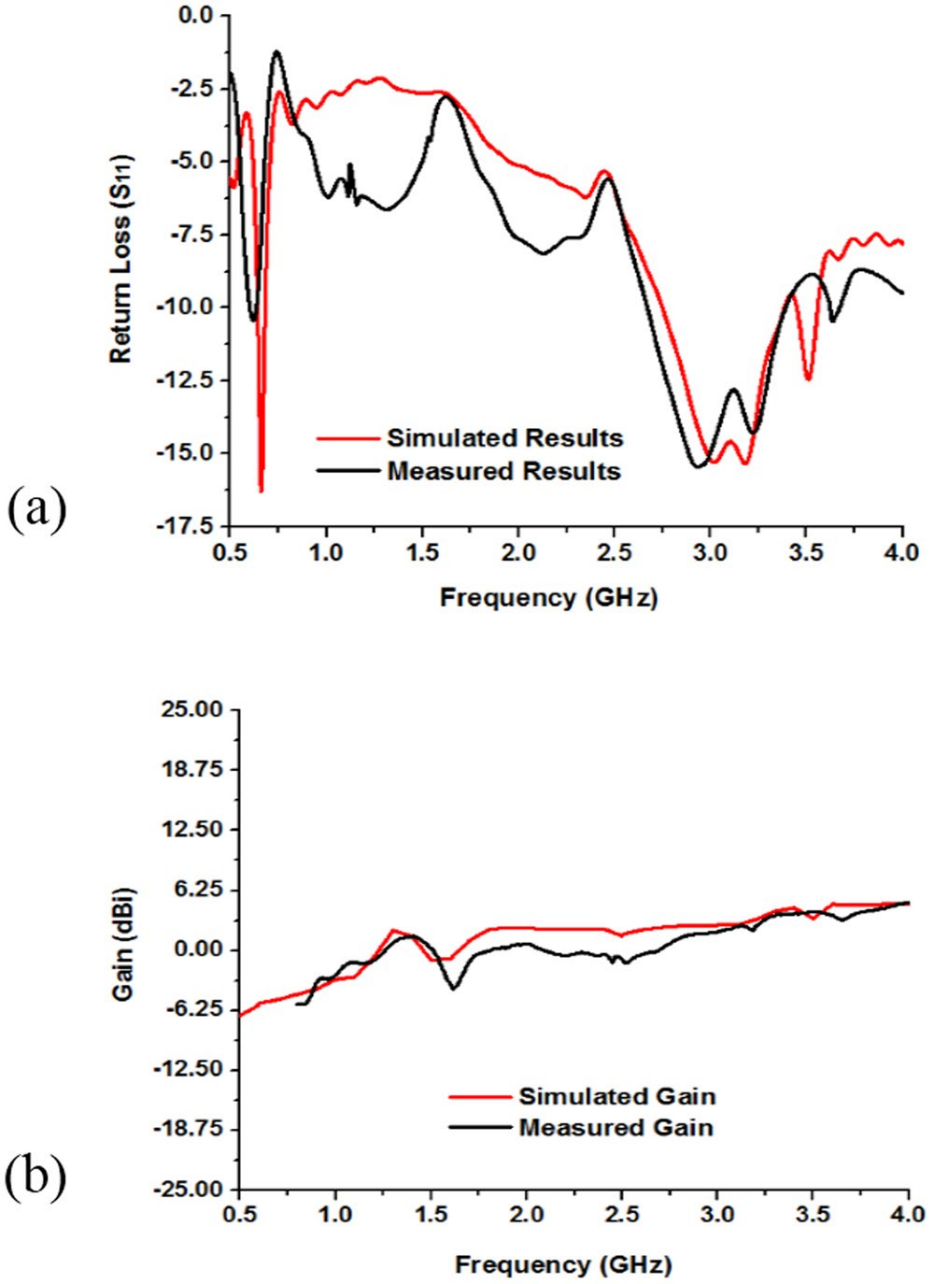
Measured and simulated: (**a**) Return loss (S_11_) and (**b**) Gain of the metamaterial embedded antenna shown in Fig. 2(c,d).

The simulated and measured results of the proposed metamaterial antenna are shown in Fig. 11. The simulated results resonance points are sequentially at 0.65 GHz, 3.18 GHz, 3.51 GHz and the operating bands over the frequency range from 0.645~0.689 GHz (bandwidth of 44 MHz), 2.75~3.38 GHz (bandwidth of 630 MHz), and 3.45~3.56 GHz (bandwidth of 110 MHz), whereas the measured results resonance points are respectively, 0.63 GHz, 3.21 GHz, 3.63 GHz that covers the bandwidth of 40 MHz (0.60~0.64 GHz), 730 MHz (2.67~3.40 GHz), and 60 MHz (3.61~3.67 GHz). The simulated results are in good accordance with the measured ones and slight discrepancies between the simulated and measured results for imperfect soldering, and fabrication tolerance. Besides, the substrate relative permittivity is an important factor for the variation of the simulated and measured results. The resonant frequency depends on the permittivity of the substrate material. If the permittivity is increased, then the resonant peaks are shifted toward the lower frequency. Furthermore, the measured gains at the dual operating frequency bands are 0.15~3.81 dBi and 3.47~3.75 dBi over the frequency range of 2.67~3.40 GHz and 3.61~3.67 GHz in Fig. 11(b). Besides, in Fig. 11(b) the measured gain frequencies are from 0.8 GHz to 4.0 GHz, whereas the simulated frequencies are from 0.5 GHz to 4.0 GHz because the measurement is done is Satimo StarLab and the StarLab can measured from 800 MHz to 18.0 GHz.

**Figure 12:**
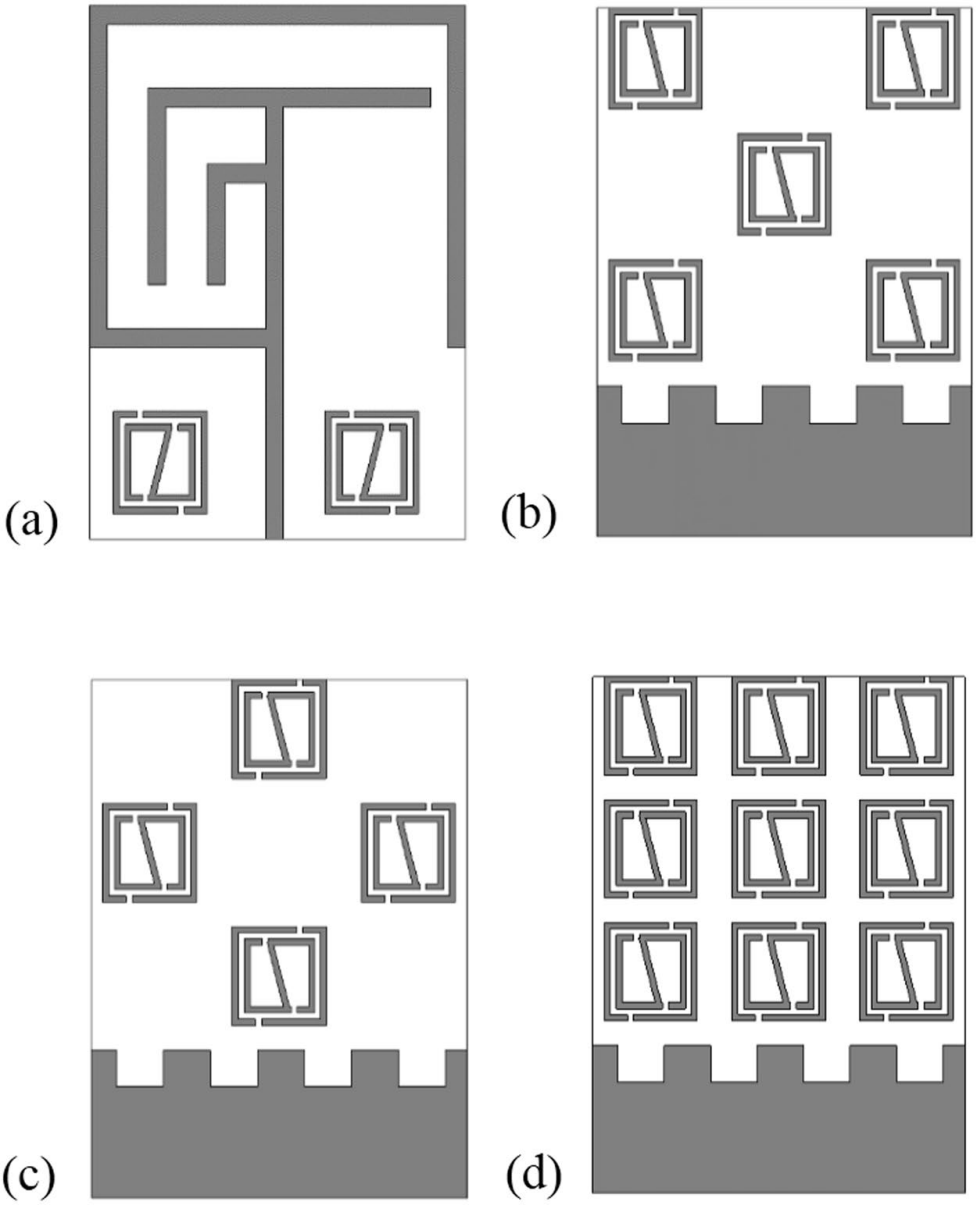
Metamaterial integration at various position of the proposed MMs antenna: (**a**) MMs antenna conf.-1, (**b**) MMs antenna conf.-2, (**c**) MMs antenna conf.-3 and (**d**) MMs antenna conf.-4.

Figure 12(a–d) display the metamaterial embedded antenna structure. In is seen in the figure the metamaterial structure is embedded in different position in the radiating patch and ground plane. In Fig. 12(a) the metamaterial structure is embedded both side of the feed line in patch structure, whereas in Fig. 12(b–d) metamaterial structure is integrated at different position in the ground plane. Metamaterial is a man-made artificial material, which has some unique electromagnetic characteristics. So, by embedding the metamaterial structure in the antenna, the antenna performance is increased and the make the antenna compact by reducing the antenna size.

**Figure 13:**
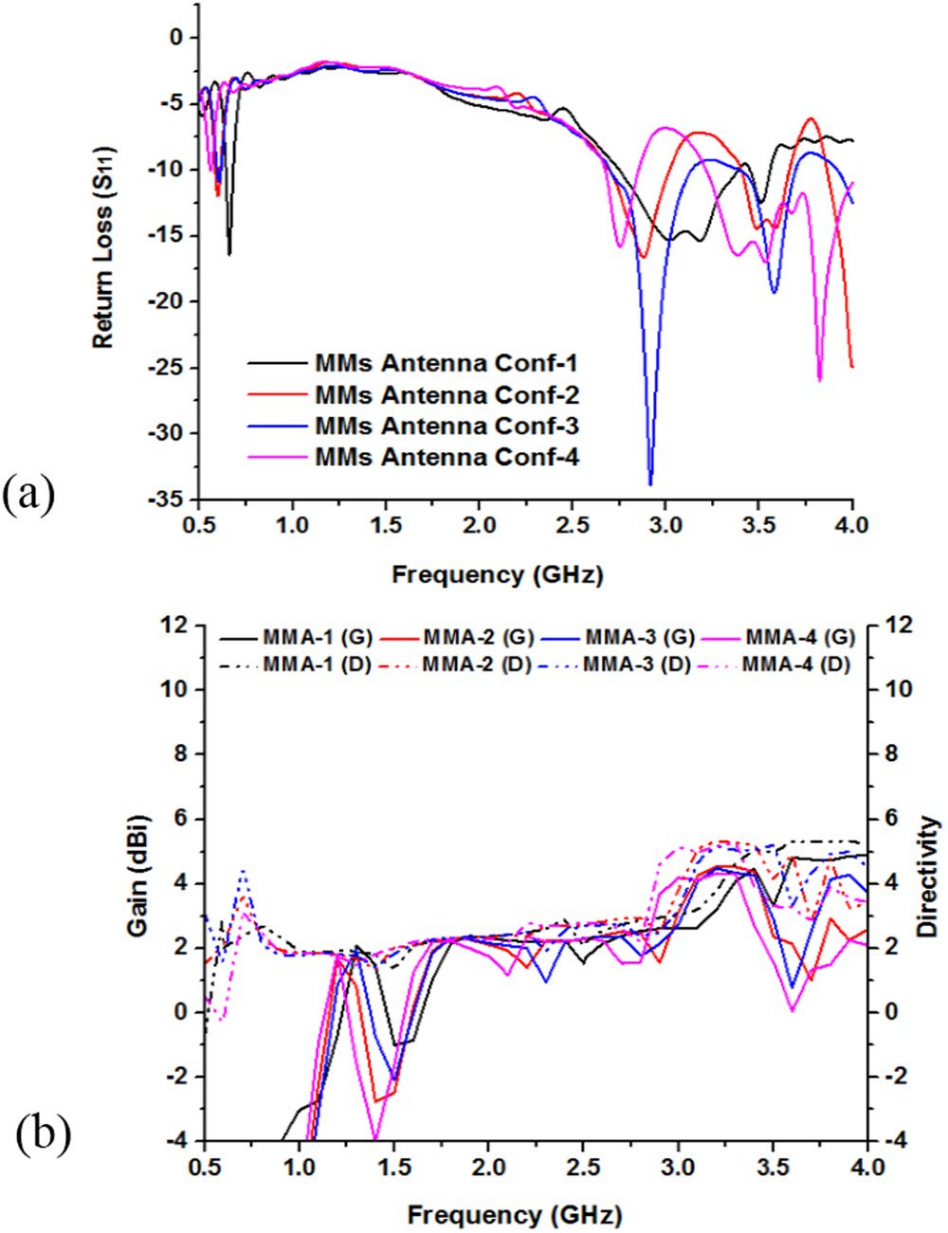
Performance analysis by integration MMs at different position of the proposed metamaterial antenna: (**a**) Return loss (S_11_) and (**b**) Gain & Directivity.

The impact on the performances for embedding the metamaterial at different position of the antenna by simulation is shown in Fig. 13(a,b) and Table 4. From Fig. 13(a), it is seen that all of the three MMs antenna configuration have covered the L-, and S-band but there are variation of the resonant points and the bandwidth. Moreover, bandwidth is the most important parameter of an antenna, it describes for an antenna in which range of frequency or ranges of frequencies it can properly radiates or receives energy. In case of the MMs antenna conf.-1, the resonance points are respectively 0.65 GHz (amplitude of −15.56 dB), 3.18 GHz (amplitude of −15.30 dB), and 3.51 GHz (amplitude of −12.45 dB), which cover the bandwidth of 44 MHz (0.645~0.689 GHz), 630 MHz (2.75~3.38 GHz), and 110 MHz (3.45~3.56 GHz), respectively. Further, MMs antenna conf.-2 achieves the dual-band operations at 0.577~0.613 GHz (bandwidth of 36 MHz), 2.696~3.026 GHz (bandwidth of 330 MHz), and 3.404~3.671 GHz (bandwidth of 267 MHz). Similarly, for the MMs antenna conf.-3 cover band are 0.585~0.617 GHz (bandwidth of 32 MHz), 2.703~3.134 GHz (bandwidth of 431 MHz), and 3.41~3.69 GHz (bandwidth of 280 MHz). Finally, MMs antenna conf.-4 archives the bandwidth of 15 MHz, 185 MHz, as well as 773 MHz, respectively, whereas the center of the resonance points are sequentially, 0.56 GHz, 2.73 GHz, and 3.83 GHz.

**Table 4:** Performances of the proposed MMs antenna at different configurations.

MMs Antenna Configurations	Resonant Frequency	Band covered	Bandwidths
MMS Antenna Conf.-1	0.65 GHz, 3.18 GHz, 3.51 GHz	L-, S-Band	44 MHz, 630 MHz, 110 MHz
MMS Antenna Conf.-2	0.59 GHz, 2.89 GHz, 3.59 GHz	L-, S-Band	36 MHz, 330 MHz, 267 MHz
MMS Antenna Conf.-3	0.60 GHz, 2.92 GHz, 3.58 GHz	L-, S-Band	32 MHz, 431 MHz, 280 MHz
MMS Antenna Conf.-4	0.56 GHz, 2.76 GHz, 3.83 GHz	L-, S-Band	15 MHz, 185 MHz, 773 MHz

Antenna gain is always related to the main lobe and is specified in the direction of maximum radiation unless indicated. From Fig. 13(b) the gain and directivity of the different configuration are observed, where the MMs antenna conf.-1 has the highest gain and the MMs antenna conf.-4 has the lowest response. In addition, MMs antenna conf.-2 as well as antenna conf.-3 are almost similar. Antenna directivity represent how much radiation pattern of the design antenna is directional. If the antenna radiates in all the directions perfectly and there is no other side lobe then the directivity of this type antenna would be 1 or 0 dBi. Moreover, directivity is a dimensionless quantity science it is the ratio of two radiation intensities. An antenna that has a narrow main lobe would have better directivity, then that one which has a broad main lobe, hence it is more directive.

**Figure 14:**
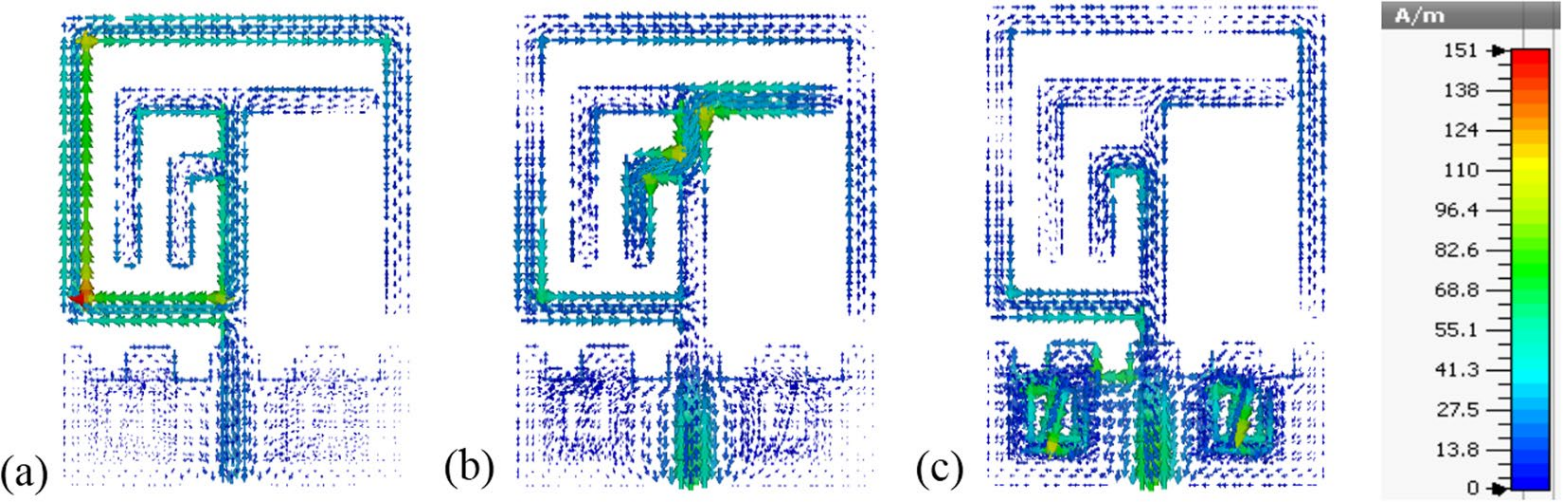
Surface Current distribution of the proposed MMs antenna at, (**a**) 0.63 GHz, (**b**) 3.21 GHz and (**c**) 3.63 GHz.

The simulated surface current distributions of the proposed antenna at 0.63, 3.21, and 3.63 GHz are shown in Fig. 14(a,b). In the figure the current distributions of the frequencies are different from each other. For the lower frequency band at 0.63 GHz, the surface currents are appeared to concentrate on the upper portion of the feed line and the larger radiator at the top edge of the patch. On the other hand, increased intensity of surface currents at higher frequency is seen in different areas other than the lower resonant mode. For 3.21 GHz frequency, the surface currents are seen to be concentrated on the bottom edge of the feed line, where the feed line and the SMA connector are connected as well as middle area of the antenna where the feed line and the “*L-shape*” like radiator are connected together. The surface current path in this case is less distributed, which lead to generation of almost homogeneous electric and magnetic fields. Finally, a much stronger current distribution is observed on the lower portion and integrated two sides metamaterial of the feed line at 3.63 GHz. Moreover, in comparison to the lower frequency the variation of the current phase along the feed line and radiator elements are clearly visible.

**Figure 15:**
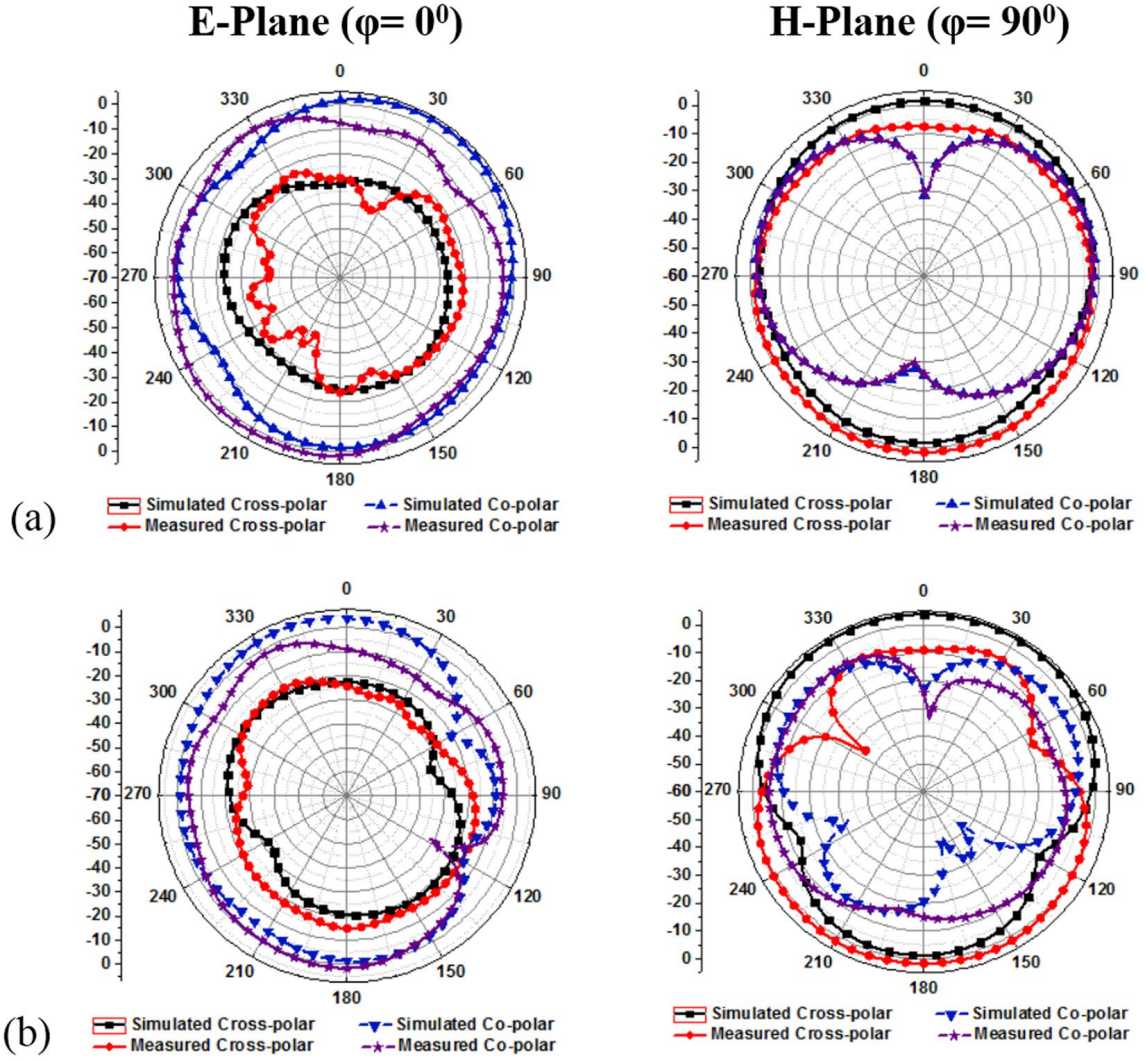
Simulated and Measured radiation pattern of proposed metamaterial inspired antenna at: (**a**) 3.21 GHz and (**b**) 3.63 GHz.

Figure 15(a,b) show the comparison between measured and simulated far-field radiation patterns in E-plane and H-plane. The figures are shown from the relevant pattern from φ = 0° (E-plane) and φ = 90° (H-plane). E_θ_ represents the co-polarization and E_φ_ defined the cross-polarization features. The xy-coordinates are taken into account as the E-plane and yz-coordinates as the H-plane. The co-polarizations is greater than the cross-polarization at 3.21 GHz and 3.63 GHz resonant frequency. Only a little deviation is found in between the measured results and the simulated results. This may happen because of the reflection in to the field between the AUT and probe. The reflection may come from the chamber scattering, antenna holder itself and the track inside the anechoic chamber. Besides, the manually calibration of the Satimo StarLab chamber machineries. Apart from that, the radiation patterns are quite impressive and show better broadside radiation features consider front to back ratio with low cross polarization, which leads to symmetric and nearly omnidirectional radiation patters.

Until present time there remain needs in the telecommunication industry to design an advance compact metamaterial antenna that overcome the shortcomings. Such metamaterial inspired antenna should ideally have a simple design and easy to practical applications. Therefore, design of compact with low profile metamaterial inspired antennas, which are capable of achieving desire high bandwidth, gain, efficiency, directivity and good radiation characteristics are still challenging task for the metamaterial antenna researchers. Table 5 illustrates the comparison between the proposed MMs inspired antenna with existing the antennas based on dimension, covered band, bandwidth, gain and applications. Moreover, the related antenna have different structure and dimension printed on the various substrate material with the different ground plane structure. As a result, the resonant frequencies are identical with the different bandwidth, gain and applications. From the table only ref.^7^ antenna is smaller in size then the proposed MMs inspired antenna but there is no application for LTE and the gains are not high. Reference^19^ has the higher gain than the proposed MMs antenna and all of the antennas existing in the table but the antenna in ref.^19^ bigger in size than all of antennas. In addition, refs^6–8^ and ref.^19^ are cover the S- and C-bands as well as applicable for Bluetooth, WLAN, WiMAX applications. Further, refs^10,14,15^ and ref.^18^ are achieved the operation of L- and S-bands, which are LTE, GPS, GSM, WLAN, and WiMAX applications. Furthermore, refs^3,5^ and refs^16,17^ are applicable for only S-band (Bluetooth, WiFi, WiMAX and WLAN) operations. Therefore, after detail investigation of the existing antennas in the Table 5 the proposed metamaterial inspired antenna is compact in size, cover lower frequency bands, moderated gain and larger bandwidths and applicable for LTE, WiMAX applications.

**Table 5:** Performance comparison between the proposed MMs antenna with the existing antennas.

References	Antenna Dimension	Resonant Frequency (GHz)	Covered Bands	Bandwidth (MHz)	Gain (dBi)	Applications
Martínez *et al*.^3^	40 × 30 mm^2^	2.40, 3.60	S-Band	230, 220	1.4, 1.7	Bluetooth, WiMAX
Li *et al*.^5^	45 × 50 mm^2^	2.25	S-Band	330	0.97	Bluetooth
Huang *et al*.^6^	45 × 40 mm^2^	2.40, 5.20	S- and C-Band	1300, 1800	3.20, 2.34	WLAN, WiMAX
Bakariya *et al*.^7^	27 × 24 mm^2^	2.40, 3.50, 5.70	S- and C-Band	85, 400, 125	1.3, 2.5, 3.8	Bluetooth, WLAN, WiMAX
Pushpakaran *et al*.^8^	40 × 38 mm^2^	2.47, 5.18	S- and C-Band	310, 560	4.5, 7.0	WLAN
Cao *et al*.^10^	44 × 56 mm^2^	1.54, 2.41, 3.25	L- and S-Band	90, 145, 700	−2, 1.52, 3.0	GPS, WLAN, WiMAX
Zhang *et al*.^14^	115 × 42 mm^2^	0.90, 1.88	L- and S-Band	376, 1757	0.8, 2.05	GSM
Liu *et al*.^15^	115 × 60 mm^2^	0.85, 1.75	L- and S-Band	271, 1125	2.5, 3.37	GSM, WLAN
Abed *et al*.^16^	70 × 50 mm^2^	2.45, 3.60	S-Band	260, 350	—	WiFi, WiMAX
Nandi *et al*.^17^	45 × 25 mm^2^	2.40, 3.50	S-Band	270, 150	−2.0, 0.14	WLAN, WiMAX
Trong *et al*.^18^	88 × 88 mm^2^	0.9, 1.70	L- and S-Band	40, 120	−0.7, 4.5	GSM
Lee *et al*.^19^	220 × 320 mm^2^	2.40, 5.20	S- and C-Band	84, 200	6.9, 6.8	WLAN
Chen *et al*.^20^	140 × 75 mm^2^	0.95	L-Band	262	—	LTE
Proposed MMs Antenna	42 × 32 mm^2^	0.63, 3.21, 3.63	L- and S-Band	40, 730, 60	3.0, 3.69	LTE, WiMAX

## Conclusion

Advancements of wireless communications and electronic warfare systems in new cutting edge technologies, include metamaterial antennas for leading the improvements in overall system performance. To adjust with the modern wireless communication systems, a metamaterial inspired antenna with covering the L- and S-band frequencies is designed, analysed and measured in this paper. Simulation results show the metamaterial inspired antenna works well in the lower (0.645~0.689 GHz) and upper (2.75~3.38 GHz) as well as (3.45~3.56 GHz) frequency range. Experimental results are very close to the simulation ones, where the covered lower and upper bands are respectively, (0.60~0.64 GHz), (2.67~3.40 GHz), (3.61~3.67 GHz). The antenna exhibits Omni-directional radiation pattern during the operating frequency band with the high peak gain. The antenna can be fabricated easily as well as cheap, simple, and compact for small portable devices. Moreover, the proposed without MMs antenna and with MMs antenna have stable radiation characteristics, high efficiency, low back lobe and good candidate for LTE, Bluetooth, WiMAX, etc. applications.
